# Identification of Statewide Hotspots for Respiratory Disease Targets Using Wastewater Monitoring Data

**DOI:** 10.3390/tropicalmed10090241

**Published:** 2025-08-28

**Authors:** Dustin Servello, Purnima Chalasani, Erica Leasure, Krysta Danielle LeMaster, Justin Kellar, Jill Stiverson, Michelle White, Zuzana Bohrerova

**Affiliations:** 1 Ohio Wastewater Monitoring Program, Ohio Department of Health, Bureau of Environmental Health and Radiation Protection, Columbus, OH 43215, USA; 2 Bureau of Public Health Laboratory, Ohio Department of Health Laboratory (ODHL), Ohio Department of Health, Reynoldsburg, OH 43068, USA

**Keywords:** wastewater, epidemiology, SARS-CoV-2, COVID-19, influenza, respiratory, spatial analysis, hotspots, Ohio

## Abstract

As wastewater monitoring networks continue to expand the monitoring of various targets, it is important to ensure these networks remain both representative of their monitored populations and flexible enough to accurately predict shifts in an expanding list of targets. In this study, we analyzed the levels of SARS-CoV-2, influenza A (InfA), and influenza B (InfB) detected in untreated wastewater during the 2023–2024 respiratory season at 70 locations participating in the Ohio Wastewater Monitoring Network. Locations with the first detection that are seasonal hotspots and sites reaching peak concentration for each target were compared and analyzed for dependence on healthcare access and population characteristics, such as population size and density, county traffic, and demographic and socioeconomic factors. The trends in these three respiratory viruses were found to closely mirror trends in clinical indicators including the number of cases and positive tests with wastewater levels providing a two-week lead for SARS-CoV-2 and no lead for influenza on these clinical indicators. InfA was first detected in more affluent sewersheds that were less racially and ethnically diverse and had higher traffic counts, while none of the parameters tested had an effect on InfB first detects. The seasonal hotspots varied for all three respiratory viruses, where InfA hotspots were exclusively in the northeast, InfB was in the southeast and east border areas, and SARS-CoV-2 wastewater hotspots concentrated around central and northwestern Ohio. While wastewater monitoring networks may not offer full coverage of all populous areas, we have shown that a spatially distributed and highly diverse network is needed for early detection of various respiratory targets.

## 1. Introduction

Since the identification of wastewater surveillance as a rapid and inexpensive tool for COVID-19 population monitoring [1], wastewater monitoring networks were established worldwide to complement COVID-19 clinical case reporting [2,3,4,5,6,7]. Wastewater surveillance provides a unique opportunity to study the spatial distribution of respiratory pathogens in a partially immune population due to the distinct infectious waves reflected in wastewater, independence of wastewater surveillance from testing and reporting bias, and inclusivity and representativeness of a broader infected population (both asymptomatic and symptomatic) [8,9,10].

Several clinical indicator studies focused on seasonal influenza and COVID-19 identified diverse drivers of infection spread, such as air travel, short-distance work commute, population density, human development index or demographic and socioeconomic factors (for example social vulnerability index SVI), green spaces, and rapid spread in populous centers followed by spread to less populous areas [11,12,13,14,15,16]. In traditional public health surveillance, the spatial distribution of reported disease might be affected either by a willingness to seek care or by limited access to healthcare or changes in disease reporting [17]. Wastewater surveillance is not dependent on these variables, since it captures everyone shedding disease markers (genetic material) in feces, urine, saliva, and sputum into wastewater during routine bathroom use. Initial research evaluating SARS-CoV-2 hotspots and spatial distribution based on wastewater was mainly performed on a neighborhood scale and at the beginning of the pandemic while there were travel restrictions and implementations of other preventative measures. Under these conditions, higher wastewater SARS-CoV-2 loads or peaks were apparent in areas with populations at greater risk for poor health outcomes [18,19,20,21] and higher population density [21]. However, some studies have also indicated that higher commercial activity, such as number of commuters [20] and higher median income [22], are associated with wastewater SARS-CoV-2 hotspots.

The initial wastewater monitoring site selection in Ohio was driven by a county-based approach to public health action with a goal of one monitoring location per county as well as prioritizing communities with those at greater risk for poor health outcomes and high COVID-19 prevalence [5]. Wastewater surveillance became a core component of public health monitoring [10], and as it is expanding to new infectious disease targets, the need for sustainable monitoring networks is emerging. There are many factors influencing site prioritization including the identification of hotspot sites that can be defined either as locations with first detection of the target (for diseases with defined season that have a period of very low detection such as influenza), locations with the earliest season onset (reaching 80th percentile prevalence), and locations with early warning potential or all-season prevalence hotspots. Determination of hotspots and evaluation of the factors influencing hotspots, such as demographic and socioeconomic factors, closeness to highway, mobility, population density, and others can assist in maintaining surveillance networks that include representative locations with potential for early warning.

In this manuscript, we identified hotspot locations in Ohio’s statewide monitoring network using wastewater monitoring over one respiratory season for three infectious disease targets—SARS-CoV-2, InfA, and InfB. This is the first time there has been an evaluation of the breath of demographic, mobility, and socioeconomic factors at the scale of a statewide wastewater monitoring network that additionally characterizes wastewater monitoring locations identified as statewide hotspots for multiple respiratory pathogen targets. This novel analysis establishes a foundation for other statewide networks to restructure their networks to maintain sustainability into the future. We intend to use these analyses together with other parameters to select core sites for long-term infectious disease wastewater monitoring.

## 2. Materials and Methods

### 2.1. Study Location Inclusion Criteria and Sample Collection

Of the 76 wastewater treatment plants (WWTP) regularly providing samples to the Ohio Wastewater Monitoring Network (OWMN), 70 plants met the inclusion criteria for this analysis. Locations were excluded for the following reasons: (i) locations that had large gaps in sampling greater than two weeks during the peak season from 1 December 2023 to 29 February 2024; (ii) locations that were missing flow rate information (submitted by the treatment plant); (iii) locations that did not detect a concentration of InfA above the level of quantification (LOQ) during the examined period; and (iv) locations that did not detect a concentration of InfB above the LOQ during the examined period. This analysis includes 3815 samples of untreated wastewater collected from the influent of 70 wastewater treatment plants from 1 October 2023 to 30 April 2024. Samples were collected twice weekly as a 24-h manual (1 location), time-weighted (27 locations) or flow-weighted (40 locations) composite or grab sample (2 locations). If a composite sample could not be collected, a grab sample was taken. This happened only for 120 out of 3815 samples (3.14% of samples). There were occasions for a given facility where the concentration of SARS-CoV-2 was below LOD. The wastewater concentration of SARS-CoV-2 was below LOD for 846 out of 3815 samples (22.2% of samples). These instances of below LOD SARS-CoV-2 occurred during the beginning (October) and end (March/April) of the season examined. Samples were delivered by courier service to the Ohio Department of Health Public Health Laboratory (ODHL) on ice to maintain a 2–8 °C temperature range within 32 h of completing composite or grab sampling.

### 2.2. Sample Concentration and Quantification of SARS-CoV-2, InfA, and InfB

Samples received at ODHL were mixed by inversion and then incubated at room temperature for 45 s to allow for gravitational separation of solids. Forty milliliters of wastewater was transferred to a sterile cup, and 400 µL of 10% Tween 20 and 50 µL of Bovine Coronavirus (BCoV) (Merck & Co, Inc., Rahway, NJ, USA) at 3553 copies/µL were added to the sample and mixed gently by inversion. Concentration and extraction were completed in duplicate for each sample.

Virus capture and concentration were carried out using the Ceres Nanotrap Microbiome A Particles (Ceres Nanosciences Inc., Manassas, VA, USA). Briefly, 50 µL ER2 and 75 µL Microbiome A Particles were added to 4.75 µL sample in two 24 deep well plates for a total of 9.5 mL concentrated sample, and concentration was carried out using the KingFisher Apex (ThermoFisher, Inc., Waltham, MA, USA). The viral bound beads were eluted into 400 µL of ThermoFisher MagMax lysis buffer.

Nucleic Acid Extraction was performed using the Qiagen AllPrep Powerviral RNA kit (Qiagen, Inc., Germantown, MD, USA) with modification to extract 96-well plates on the Qiacube HT. We added 200 µL of eluted sample concentrate to 600 µL PM1 and 6 µL β-mercaptoethanol in a Qiagen S block. Samples were then vortexed for 30 s and incubated at room temperature for 5 min. IRS (150 µL) was then added to each sample and vortexed to homogenize and incubated at 4 °C for 5 min. The S block was centrifuged at 13,000 times the force of gravity (g) for 1 min at 4 °C. A custom program was provided by Qiagen to carry out the remaining steps of the AllPrep PowerViral protocol on the Qiagen HT. The QIAAMP 96-well filter plate was used in lieu of the filter columns.

The Qiagen Qiaquity digital PCR platform was used to quantify SARS-CoV-2, InfA, InfB, and BCoV. BCoV was quantified as an internal control to estimate viral recovery. The GT-Molecular kit for Qiaquity (GT-Molecular, Ft Collins, CO, USA) was used in conjunction with the Qiagen Advanced Probe kit for quantification of SARS-CoV-2 (SC2 nucleocapsid gene), InfA (both InfA1 and InfA2 combined), InfB, and BCoV. Quantification was carried out on 24-well nanoplates with 26 k partitions per well. Limit of quantification, LOQ, for SARS-CoV-2, InfA, and InfB was 13,000 copies/L. For SARS-CoV-2, for both types of non-detects where there was either no amplification or levels below the LOQ, a value of 6500 (LOQ/2) was imputed. For InfA and InfB, if a replicate for a sample was not amplified, a value of 0 was imputed. If a replicate amplified but was below LOQ, half of the LOQ (6500) was imputed. The average of the replicates was used for analyses.

### 2.3. Supplemental Data Collection and Preparation

(i) Wastewater and Clinical Data

For wastewater data, the concentration (gene copies per liter) of the three targets was normalized by multiplying them by the flow rate provided by the WWTP as average flowrate over the 24-h composite or the flowrate at the time of the grab sample. The flow normalized concentrations were rounded and divided by 1,000,000 to obtain million gene copies per day. The million gene copies per day concentrations were then divided by the population in the sewershed estimated by the WWTP to obtain a flow-population normalized concentration in million gene copies per person per day (MGCPD).

COVID-19 cases were identified from the Ohio Disease Reporting System (ODRS) and extracted by the Innovate Ohio Platform (IOP) using the sewershed boundaries defined by a GeoJSON composed of shapefiles provided by the WWTPs. Shapefiles were generated in ArcMap 10.7 (ESRI) from sewer network maps, pipework, or municipal boundaries for WWTPs that did not have a sewershed shapefile readily available. The COVID-19 cases were extracted specifically for monitored areas and include suspected, probable, and confirmed cases by PCR and antigen tests or at home tests. For COVID-19 positive tests, the number of positive COVID-19 tests by week from public health laboratories and selected clinical laboratories participating in the National Respiratory and Enteric Virus Surveillance System (NREVSS) in the state of Ohio were used. The NREVSS COVID-19 data were used in addition to the COVID-19 cases so that there were comparable clinical metrics between COVID-19 and influenza. For influenza-positive tests (InfA and InfB individually), the number of positive influenza tests by week by serotype from NREVSS were used.

(ii) Demographic and Infrastructure Data

Data regarding the population served by the WWTP were provided by the WWTPs. The sewershed area (km^2^) was calculated from sewershed shapefiles that were provided by the WWTP or generated using sewer networks, pipework, or municipal boundaries in ArcMap 10.7 (ESRI). Population density was calculated from the population served and sewershed area as the number of persons per km^2^. The total number of hospitals, hospital beds, urgent care facilities, and nursing homes or assisted living facilities within a sewershed were determined through intersects of sewershed shapefiles and shapefiles for public use provided by the Geospatial Management Office (Department of Homeland Security (DHS) Homeland Infrastructure Foundation-Level Data (HIFLD) database) in ArcMap 10.7. Demographic and socioeconomic factor data for census tracts composed of the overall SVI as well as SVI for four themes (socioeconomic status, household characteristics, racial and ethnic minority status, and housing type and transportation) for a given sewershed were determined using an intersect (ArcMap 10.7) of sewershed shapefiles and census tract datasets prepared by the Agency for Toxic Substances and Disease Registry (ATSDR) within the Centers for Disease Control and Prevention (CDC, Department of Health and Human Services). The demographic and socioeconomic factors data were collected by the 2020 American Community Survey (U.S. Census Bureau, Department of Commerce). Sewershed distance from primary roads (interstate highways or state routes) was determined in ArcMap 10.7 by intersect of three buffers (5, 10, and 30 mi) generated around sewershed boundaries and polyline information from publicly available shapefiles from the U.S. Census Bureau. Data regarding county level annual average daily traffic were collected from Ohio Department of Transportation (ODOT) traffic stations.

### 2.4. Data Analyses

(i) SARS-CoV-2 and Influenza Sewershed Ranks and Comparisons

Dates were recorded for when each location had their first above LOQ detection of InfA or InfB, reached the 80th percentile SARS-CoV-2, InfA, or InfB wastewater concentration for the location during the analyzed period, or reached their peak SARS-CoV-2, InfA, or InfB wastewater concentration. The first above LOQ detection of SARS-CoV-2 could not be ranked, since SARS-CoV-2 concentrations were not below LOQ at most locations throughout the monitoring period. Percentiles were determined by a proc univariate statement in Statistical Analysis Software (SAS 9.4, SAS Institute). The maximum concentration for the period for each location was determined in SAS by a proc sql statement. Locations were ordered from the earliest to latest for each of the parameters using total preorder method, and ties were resolved using fractional ranking—the average of the rankings for locations with the same rank was used. For example, if three locations shared the rank of 1 (ordered as 1, 2, and 3 in the order of locations), the ranking would be calculated from the average [(1 + 2 + 3)/3 locations] making the rank for each of the three locations 2. A Kruskal–Wallis H Test (SAS) was used to determine the statistical significance among the ranks. A *p*-value < 0.05 indicated that the rank among the locations was not random, and that facility location had a significant effect on the rank value. The difference in days from the first above LOQ detection of InfA or InfB for each location to the location’s peak InfA or InfB wastewater concentration was also calculated.

(ii) Hotspot Cluster and Outlier Analyses

A Getis-Ord Gi* analysis in ArcMap 10.7 of the average wastewater concentration from 1 October 2023 to 30 April 2024 for each location alongside a spatial weight matrix was used to determine hot or cold spots for each of the targets (SARS-CoV-2, InfA, and InfB). The spatial weights matrix was calculated for the 70 WWTP using a K nearest neighbor (8 neighbors) conceptualization of the spatial relationships. A cluster/outlier analysis (Anselin Local Moran’s I) was also conducted using the average wastewater concentration for the period and the spatial weights matrix.

(iii) Pairwise Spearman Correlations

A set of three normality tests (Kolmogorov–Smirnov, Cramer–von Mises, and Anderson–Darling) conducted in SAS using the proc univariate statement determined that the wastewater concentration data were not normally distributed (*p* < 0.05). As the data did not fit a normal distribution, correlations between wastewater data and clinical indicators (COVID-19 cases and positive influenza tests) were determined by Spearman correlation conducted in SAS using the proc corr statement. Additionally, similar correlations where the clinical indicators were lagged by one and two weeks relative to wastewater data were also conducted. Spearman correlations were used for the normalized rankings of locations based on first detection, reaching the 80th percentile wastewater concentration, or reaching peak wastewater concentration of SARS-CoV-2, InfA, or InfB as dependent variables and sewershed population, population density, demographic and socioeconomic factors (overall, socioeconomic status, household characteristics, racial and ethnic minority, and housing type and transportation SVI), average daily traffic, and number of healthcare centers (numbers of hospitals, hospital beds, urgent care centers, and nursing homes or assisted care facilities) acting as independent variables. Spearman correlations between the listed parameters and the z-scores calculated from the Getis-Ord Gi* analysis were also calculated. Correlations with *p*-values < 0.05 were considered significant.

(iv) Cross-Correlation and Forward Stepwise Multiple Regression

A Pearson cross correlation analysis was conducted for the independent data that fall into the following three categories: population metrics (population served, sewershed population density, and county traffic), access to healthcare (number of hospitals, number of hospital beds, number of urgent care centers, and number of nursing homes or assisted living facilities), and demographic and socioeconomic factors (overall SVI, socioeconomic SVI, household characteristics SVI, racial and ethnic minority SVI, and housing type/transportation SVI).

A multiple correlation coefficient was calculated using a forward, stepwise multiple regression model by proc reg in SAS. Variables representing population, healthcare access, and demographic and socioeconomic factors were selected for the model based on these variables having lower, non-significant cross-correlation coefficients between them while also highly correlating with other variables in their category (Table 1). For example, from population metrics, sewershed population density was selected, as it was highly correlated with population served and county traffic but not correlated with the variables selected from healthcare access and demographic and socioeconomic category. Similarly, for healthcare access, the number of urgent care centers was used. For demographic and socioeconomic factors, the household characteristics SVI was used. The multiple regression model included a dependent variable represented by the normalized ranking or z-score from spatial analyses and the following three independent variables: sewershed population density, number of urgent care centers, and household characteristics SVI. Multiple correlation coefficients with *p*-values < 0.05 were considered significant. In order for a model to be generated, the addition of a given parameter must meet an R^2^ > 0.15 cutoff.

## 3. Results

### 3.1. Comparison of Statewide Wastewater Concentrations to Clinical Indicators

**Figure 1:**
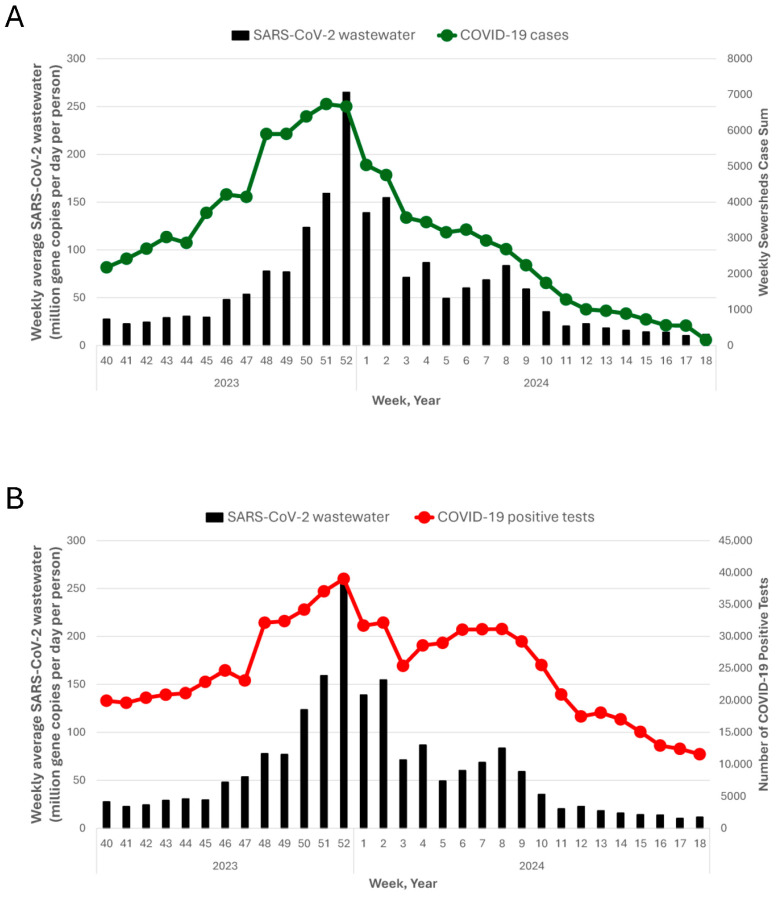

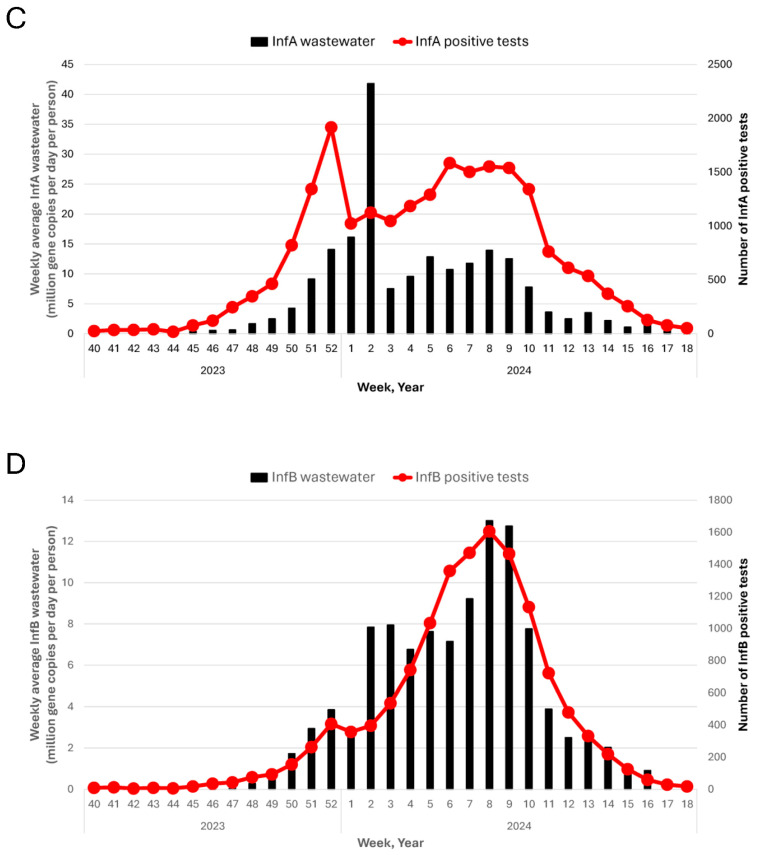
Statewide average SARS-CoV-2 wastewater concentration (million gene copies per day per person) compared to (**A**) sum COVID-19 cases by week, (**B**) number of COVID-19 positive tests by week, (**C**) sum InfA positive tests by week, (**D**) sum InfB positive tests by week.

There were two periods of increased levels of SARS-CoV-2 in wastewater in Ohio during the period analyzed, with the first occurring week 46 of 2023 to week 5 of 2024 (13 November 2023 to 4 February 2024) and a second smaller increase occurring week 6 of 2024 to week 10 of 2024 (12 February 2024 to 10 March 2024; Figure 1A). Similar trends were seen for COVID-19 cases, COVID-19 positive tests, and SARS-CoV-2 wastewater (Figure 1B). The level of SARS-CoV-2 in wastewater was highly correlated with both COVID-19 cases (0.88, *p* < 0.0001) and COVID-19 positive tests (0.96, *p* < 0.001). There was an increase in the correlation between wastewater and cases when cases were lagged by one (0.93, *p* < 0.0001) or two (0.96, *p* < 0.0001) weeks relative to wastewater. On the other hand, the correlation between wastewater and positive tests was highest with no lag between these data when lagging positive tests by one (0.90, *p* < 0.001) or two weeks (0.86, *p* < 0.001) relative to wastewater resulted in lower correlations. The difference in the wastewater predictability between these two clinical parameters is not surprising, since COVID-19 cases include nonspecific data (suspected, probable, confirmed cases) specifically extracted for monitored areas, while COVID-19 positive tests are collected as a statewide metric from only participating laboratories.

The rates of detection for InfA and InfB in wastewater samples above LOQ were 18% (669/3815 samples) and 10% (393/3815 samples), respectively. Both the InfA and InfB waves began in week 50 of 2023, but while the InfA wave subsided in week 11 of 2024, the InfB wave continued into week 14 of 2024 (1 April 2024 to 7 April 2024; Figure 1C,D). Significant correlations were seen for the level of InfA and InfB in wastewater and InfA and InfB positive tests (0.94, *p* < 0.0001 and 0.95, *p* < 0.0001, respectively) with the strongest correlation at no lag in positive tests compared to wastewater.

### 3.2. Locations That Were First to Detect InfA and InfB in Wastewater

Influenza is a seasonal respiratory disease with typical onset, peak, and duration during winter and spring. Since influenza detection in wastewater is more challenging due to lower human fecal shedding, the wastewater influenza detection above level of quantification might indicate influenza season onset. Wastewater levels of InfA and InfB above LOQ were first detected on 3 October 2023 and 26 November 2023, respectively. For InfA, 25% of locations (18 facilities) had detected a level of InfA in wastewater above LOQ by week 50 of 2023 (18 December 2023) which was four weeks prior to peak levels in wastewater (Figure 1C). For InfB, 25% of locations had detected a level of InfB in wastewater above LOQ by week 52 of 2023 (26 December 2023), which was eight weeks prior to the InfB peak in wastewater (Figure 1D).

**Figure 2:**
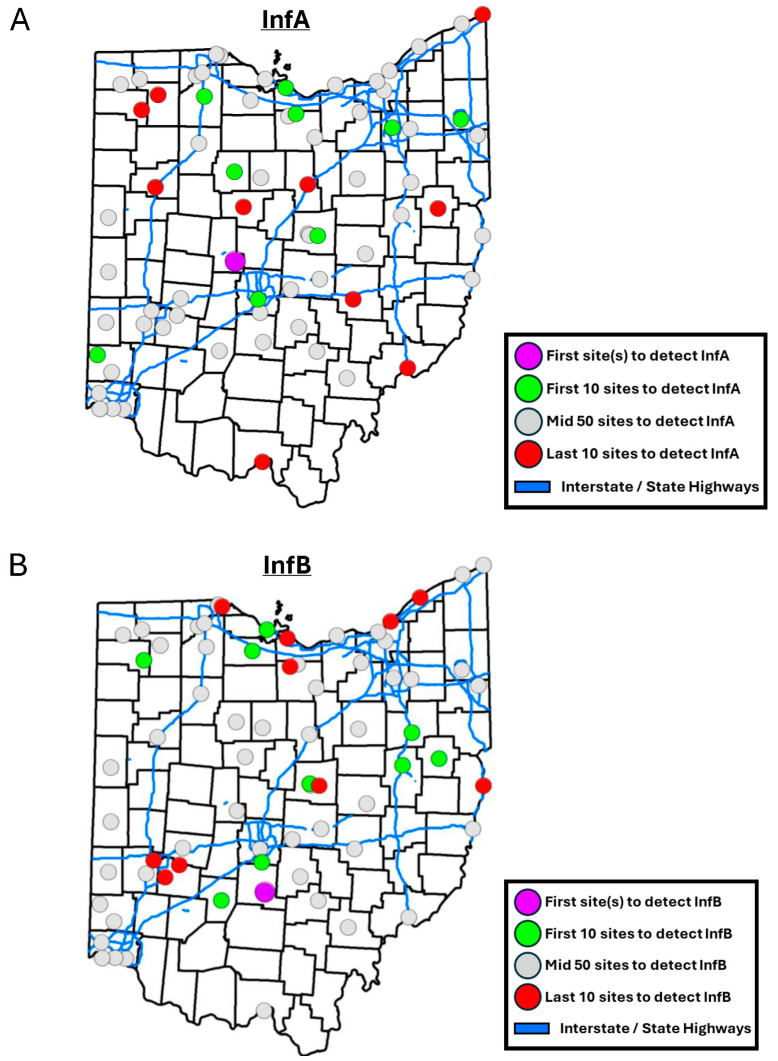
Map indicating the ranking groups of locations based on when they first detected (**A**) InfA. (**B**) InfB.

During the one season evaluated, locations that were the earliest to detect an above LOQ level of InfA tended to be geographically located mainly in the north region of Ohio along the coast and surrounding areas of Lake Erie (Figure 2A). There was not a strongly discernible, localized pattern for locations detecting InfB at an earlier time (Figure 2B). The first locations with InfA and InfB detection above LOQ were both in the central region in small communities next to a large city (Figure 2A,B). Pairwise Spearman correlations between location detection ranks and independent variables showed locations that were the earliest to detect InfA in wastewater tended to be in counties of the state with higher amounts of car traffic (−0.26, *p* < 0.05) and were located in sewersheds with lower overall SVI (0.27, *p* < 0.05), household characteristics (0.38, *p* < 0.01), and housing type and transportation SVI (0.24, *p* < 0.05). The sewershed distance to highways, size of population, population density nor the number of hospitals, hospital beds, urgent care and nursing homes, or other demographic and socioeconomic factors had an effect on the rank of detection of InfA. A forward, stepwise multiple regression model composed of the normalized location InfA detection ranking, sewershed population density, number of urgent care centers, and household characteristics SVI was generated. The residuals of the model generated had a mean around 0 and were normally distributed. The first step of the model integrated household characteristics SVI and resulted in a multiple correlation coefficient of 0.38 (*p* < 0.01). Second step addition of the number of urgent care centers resulted in a multiple correlation coefficient of 0.44 (*p* < 0.001). The addition of sewershed population density did not significantly improve the model generation. The final model fits the following equation:

normalized ranking = 56.3 (household characteristics SVI) − 1.80 (number of urgent care centers) + 9.21 (1)

While InfA was first detected in sewersheds with lower SVI, InfB was detected earlier at locations within communities with higher housing and transportation SVI (−0.37, *p* < 0.01) and lower racial and ethnic minority status SVI (0.24, *p* < 0.05). A forward, stepwise multiple regression model composed of the normalized location InfB detection ranking, sewershed population density, number of urgent care centers, and household characteristics SVI was not able to be generated, as none of the parameters met the minimal significance threshold for model generation.

### 3.3. Locations Reaching High Prevalence of SARS-CoV-2, InfA, and InfB Earlier Using 80th Percentile Concentration Threshold

Previous work by other groups in modeling the seasonal onset for respiratory viruses has used a mix of frameworks focused on reaching a percentile or incidence in cases or other clinical indicators [23,24,25,26]. We used an 80th percentile wastewater concentration threshold to rank sites that first reached a high prevalence based on the locations’ historical concentrations during SARS-CoV-2, InfA, and InfB waves. Pairwise Spearman correlation analyses did not show any significant trend in locations that showed earlier vs. later high wastewater prevalence (as indicated by the 80th percentile of concentration) in terms of the demographic and infrastructure variables tested (−0.14–0.23, *p* = 0.06–0.99, Table 1) for any of the infectious disease targets. A forward, stepwise multiple regression model could also not be generated for any of the three targets as none of the parameters met the minimal significance threshold for model generation. A Kruskal–Wallis H test of these rankings (*n* = 3 for each location) indicated that locations were not significantly more likely than others to have detected an 80th percentile threshold for a respiratory target relative to other locations indicating that the rankings could not be ruled out as random chance (*p* = 0.28).

### 3.4. Locations Identified as Seasonal Hotspots and with Earliest Concentration Peaks of SARS-CoV-2, InfA, and InfB

**Figure 3:**
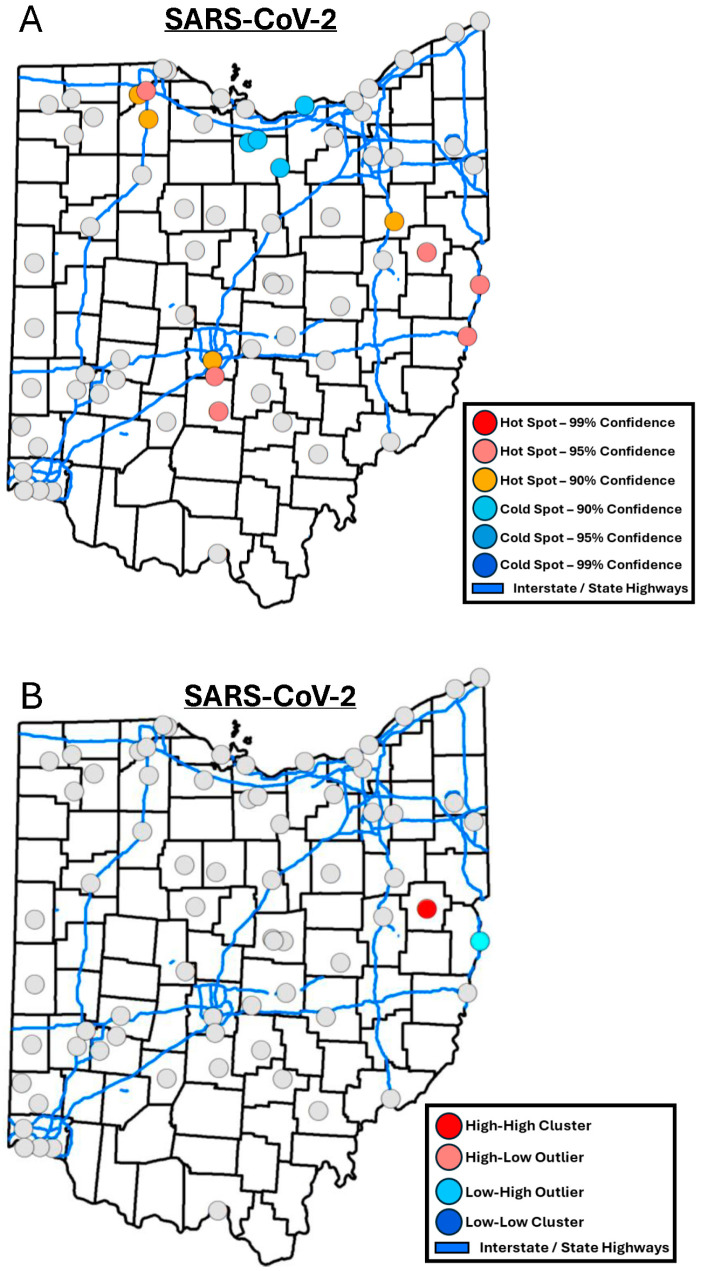
(**A**) Map of SARS-CoV-2 wastewater hotspots determined by Getis-Ord Gi* analysis. (**B**) Map of SARS-CoV-2 wastewater spatial clusters and outliers determined by Local Anselin Moran’s I analysis.

In Ohio, spatial analysis identified 10 locations as hotspots and 4 locations as cold spots for SARS-CoV-2 (Figure 3A). One of the locations identified as a hotspot was also identified as a high–high location that represented a significant cluster of high values (Figure 3B). When analyzing the pairwise Spearman correlation between the z-scores generated from the Getis-Ord Gi* analysis (higher score indicating hotspot) and demographic and infrastructure data, a significant positive correlation was found between z-score and the number of urgent care facilities in the sewershed (0.24, *p* < 0.05). Locations were ranked based on when they reached their peak SARS-CoV-2 concentration in wastewater. Spearman correlations indicated that locations within sewersheds with a higher number of urgent care facilities (−0.26, *p* < 0.05) and a higher housing type and transportation SVI (−0.31, *p* < 0.01) tended to reach their peak in SARS-CoV-2 wastewater concentration earlier.

**Figure 4:**
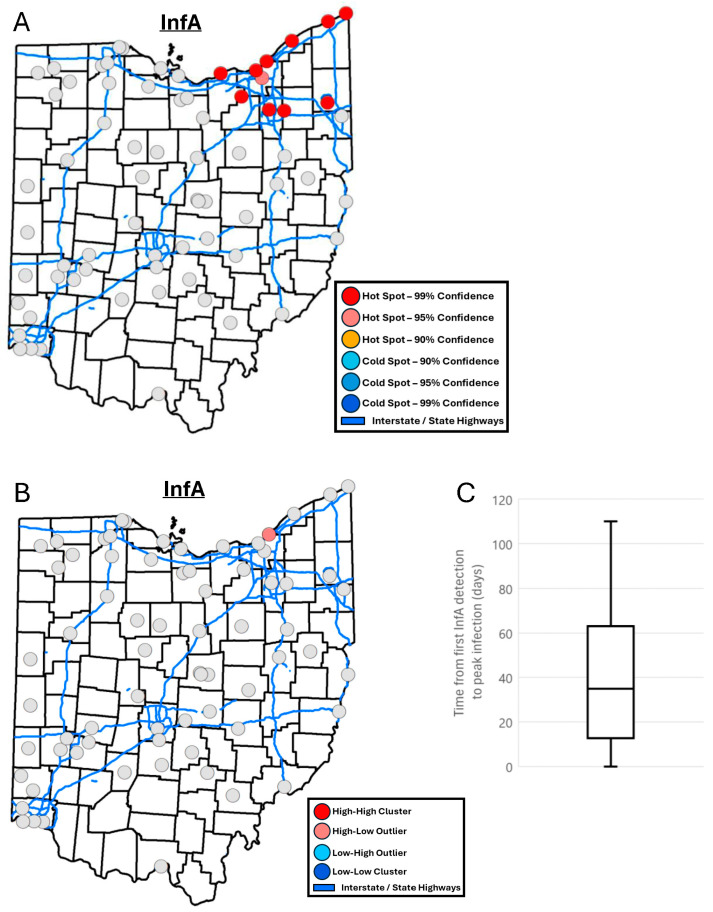
(**A**) Map of InfA wastewater hotspots determined by Getis-Ord Gi* analysis. (**B**) Map of InfA wastewater spatial clusters and outliers determined by Local Anselin Moran’s I analysis. (**C**) Box and whisker plot of the difference in the number of days between when a location first detected InfA and when it reached its peak InfA concentration.

A hotspot analysis of InfA concentration in wastewater identified all locations in the northeastern region of Ohio as hotspots (Figure 4A). One of these locations was identified as a high–low outlier within the region indicating that, even among the other hotspot locations in the region, this location was an outlier with a high concentration of InfA (Figure 4B). A correlation analysis between Getis-Ord Gi* z-scores and demographic and infrastructure data indicated a significant positive correlation with population served (0.29, *p* < 0.05), racial and ethnic minority status SVI (0.28, *p* < 0.05), sewershed area (0.26, *p* < 0.05), and county level car traffic (0.24, *p* < 0.05). No significant correlations were identified between rankings of when locations reached peak InfA in wastewater and demographic and infrastructure data (−0.04–0.12, *p* = 0.34–0.95). The average number of days between a location detecting InfA for the first time during the examined period and reaching its peak InfA wastewater concentration was 37 ± 29 days (Figure 4C).

**Figure 5:**
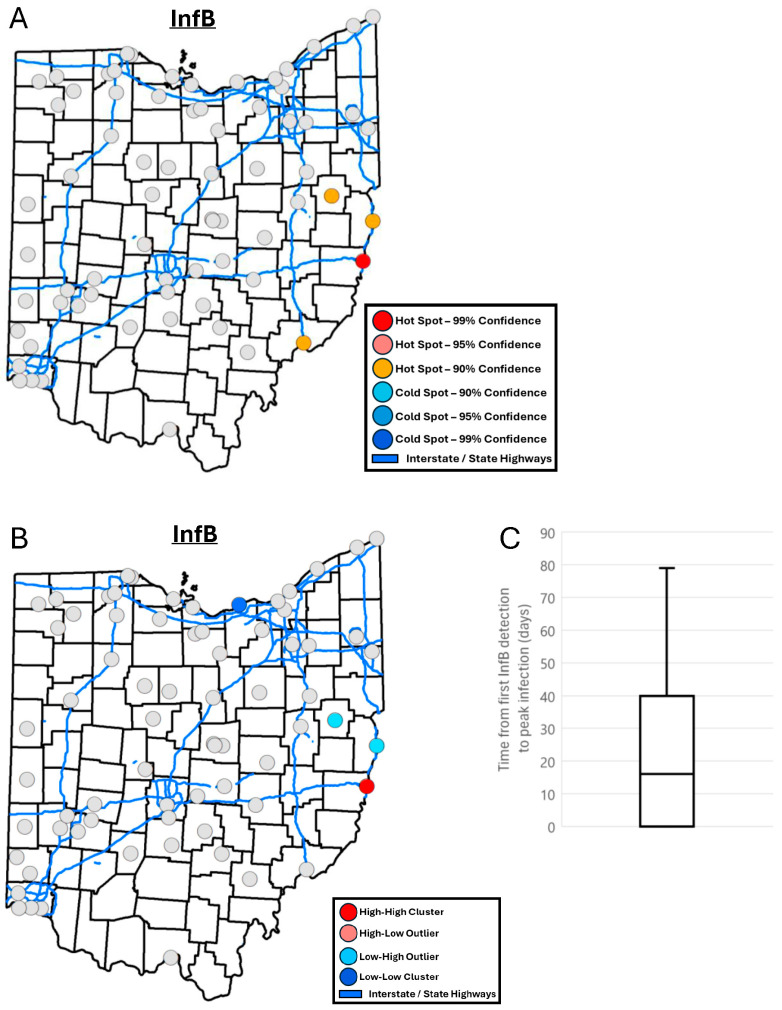
(**A**) Map of InfB wastewater hotspots determined by Getis-Ord Gi* analysis. (**B**) Map of InfB wastewater spatial clusters and outliers determined by Local Anselin Moran’s I analysis. (**C**) Box and whisker plot of the difference in the number of days between when a location first detected InfB and when it reached its peak InfB concentration.

A spatial analysis of InfB in wastewater identified four locations as hotspots. Three of these locations were on the east and southeast border of Ohio (Figure 5A). One of these hotspots was further identified as a high–high location that represents a cluster of high InfB concentration values (Figure 5B). Z-scores from Getis-Ord Gi* analysis were shown to negatively correlate with racial and minority status SVI (−0.26, *p* < 0.05) indicating that sewersheds with lower racial and ethnic diversity tend to be hotspots for InfB. Locations were ranked based on when they reached their peak InfB concentration in wastewater. Spearman correlation indicated that locations within sewersheds with a higher housing type and transportation SVI tended to reach their peak InfB wastewater concentration earlier (−0.25, *p* < 0.05). The average number of days between a location detecting InfB for the first time during the examined period and reaching its peak InfA wastewater concentration was 24 ± 24 days (Figure 5C).

A forward stepwise multiple regression model could not be generated for any of the three targets, as none of the parameters met the minimal significance threshold for model generation.

## 4. Discussion

The comprehensiveness of wastewater monitoring networks is an important factor for ensuring the accurate representation of the monitored population, while also maximizing the potential for the early detection of circulating respiratory and other diseases. Here, we find that maintenance of a spatially diverse network containing sewersheds that vary in location, access to healthcare centers, and demographic and socioeconomic factors is a highly optimal approach for early detection and quantification of three respiratory disease targets.

We found a high level of heterogeneity in the profiles of locations that were more likely to first detect the different respiratory targets prior to seasonal onset or were hotspots for these targets. In our study, InfA was first detected in more affluent sewersheds that were less racially and ethnically diverse and had higher traffic counts. This supports previous work from other groups that indicates InfA spread is promoted in areas with a greater flow of people and high socioeconomic activity [11,16,27,28,29]. A mobility study in Ohio confirmed that people from higher social economic status engage in more diffuse long-distance travel [30]. Additionally, several researchers reported a relationship between influenza risk ratio or spread and distance from interstate highways [12,31]. However, our study did not show a direct association between sewershed distance from interstate highways and the first detection or presence of InfA and instead showed a direct association with county-level traffic activity.

During the analyzed respiratory season, InfA in wastewater peaked during the first week of January, while InfB peaked in mid-February, although both of the influenza waves overlapped for a significant time of the season. Despite similar mechanisms of transmission, InfB hotspot locations differed geographically from InfA hotspot locations and tended to be on the southeastern and eastern border of the State. Locations that were the first to detect or were hotspots for InfB showed a weak association with most of the tested variables. Other groups have shown that the factors that often affect InfA epidemics have slightly altered effects on InfB. While the two have a similar seasonality, they tend to have a higher prevalence among the populations of individuals of different demographics based on age, race, and ethnicity [32,33,34,35].

SARS-CoV-2 spatial hotspots concentrated around central and northwestern Ohio and showed a very different pattern when compared to InfA hotspots that were exclusively in the northeastern part of the State. Interestingly, some SARS-CoV-2 hotspots were identical to the InfB hotspots in border towns in east Ohio. Ohio has three main airport hubs in its largest cities: Columbus (central Ohio), Cleveland (northeast Ohio), and by Cincinnati (southwest Ohio) in Newport Kentucky. Therefore, disease importation via long distance travel as indicated in previous research for COVID-19 [36,37,38] would be expected in all three of these areas and not only in central Ohio; however, our analysis determined that the areas directly around Cleveland and Cincinnati were not hotspots for this past winter seasonal wave. Similarly, large universities are present in several network locations, and no clear pattern emerged when considering student populations and SARS-CoV-2 or influenza hotspots. SARS-CoV-2 hotspots tended to correlate with a higher number of urgent care facilities, which are correlated with other healthcare access indicators, such as the number of hospitals in the sewersheds. A higher number of healthcare centers within a sewershed could mean more infected individuals traveling to these sewersheds for care possibly resulting in higher quantification in wastewater. The first detections of SARS-CoV-2 could not be calculated, due to low levels of SARS-CoV-2 being present throughout the whole year in wastewater; therefore, we evaluated the date that locations reached their peak wastewater SARS-CoV-2 concentration. This analysis also indicated that locations with an earlier peak had more urgent care facilities and were in communities with more crowding or lower rates of vehicle ownership that could result in a higher use of public transport; although whether the use of public transportation results in additional risk for transmission or infection is unclear [39,40,41]. Recent research suggests that specifically the movement of people between work and home is an important explanatory factor in the spread of COVID-19 [42,43].

When the three targets are all considered, this analysis indicates that a focus on the inclusion of population or transport centers alone in a wastewater monitoring network is likely to miss high prevalence hotspots or early detection locations for one or more of the respiratory targets examined. Furthermore, our 80th percentile target analyses indicated that once the respiratory virus is prevalent in the monitored sewersheds, the statewide locations become more homogenous and potentially less locations could be monitored to provide an accurate picture of the epidemic’s progress.

It is important to note several shortcomings of wastewater monitoring, especially related to the location where people contribute to wastewater, which might not always be where they live. An individual’s contribution to the monitoring network can be during work, travel, and when seeking healthcare, while clinical indicators and community demographic and socioeconomic factors are reported based on home address. The mean commute distance in the USA has been found to be roughly 12 miles [44], which for a network of our composition, may exceed the area of smaller sewersheds with <10,000 residents. Additionally, wastewater monitoring can indicate disease community prevalence that might not be reflective of a higher risk of disease outcomes, which are often related to those at greater risk of poor health outcomes.

Although not a main goal of our manuscript, the results also showed an interesting difference between wastewater predictability for different targets (influenza vs. COVID-19) and different data sources (COVID-19). Although sewershed-specific COVID-19 case data showed the highest correlation with a two-week lag to wastewater, the statewide COVID-19 NREVSS positive test best correlated with no lag. For influenza, only national NREVSS data were used and also showed the highest correlation with wastewater with no lag. The predictability of wastewater trends and clinical indicators should continue to be studied and seems to be affected by the clinical data classification and specificity of the clinical data for the wastewater monitored area.

While wastewater monitoring networks may not offer full coverage of all populous areas, we have shown that a network with broad healthcare access, wide spatial distribution, and varied demographic and socioeconomic population coverage is effectively predictive of clinical indicators. A network with these characteristics can provide early detection of waves of SARS-CoV-2, InfA, and InfB and contains hotspots for accurately quantifying these three targets.

## 5. Conclusions

Wastewater monitoring experienced rapid expansion and adaptation during the COVID-19 pandemic and many states, including Ohio, established large statewide networks. To manage sustainable wastewater monitoring networks, researchers and practitioners are exploring different methodologies to determine the number and location of monitoring sites. In this manuscript, we used spatial hotspot analyses and simplified rank analyses of two respiratory disease targets together with location-specific demographic and socioeconomic indicators to characterize essential locations for statewide wastewater monitoring. This manuscript contributes to ongoing research in the field, showing that statewide hotspots may be different for different targets and that the location of hotspots may be governed more by mobility than by socioeconomic status. Our analysis encompasses one respiratory season in one state for two respiratory targets and further analyses will be needed in order to continue to understand the spread and trends on larger scales and optimize monitoring location selection for these diseases. Finally, this analysis provides foundational work in guiding the identification of core sites for sustainable long-term wastewater monitoring.

## Figures and Tables

**Table 1:** Pearson cross-correlation matrix for various population-based, healthcare access, demographic, socioeconomic, and infrastructure data.

	Population Served	Population Density	County Traffic Counts	Number of Hospitals	Number of Hospital Beds	Number of Urgent Care Centers	Number of Nursing Homes/Assisted Living Facilities	Nocioeconomic Status SVI	Household Characteristics SVI	Minority and Ethnicity Status SVI	Housing Type and Transportation SVI	Overall SVI
Population served		0.34 *	0.74 *	0.92 *	0.84 *	0.87 *	0.94 *	0.20	0.09	0.6 *	−0.18	0.15
Population density	0.34 *		0.4 *	0.36 *	0.45 *	0.30	0.18	0.48 *	0.13	0.49 *	0.11	0.4 *
County traffic counts	0.74 *	0.4 *		0.74 *	0.72 *	0.67 *	0.69 *	0.12	−0.03	0.67 *	−0.34 *	0.03
Number of hospitals	0.92 *	0.36 *	0.74 *		0.93 *	0.7 *	0.88 *	0.23	0.13	0.67 *	−0.19	0.20
Number of hospital beds	0.84 *	0.45 *	0.72 *	0.93 *		0.59 *	0.77 *	0.27 *	0.13	0.66 *	−0.14	0.24
Number of urgent care centers	0.87 *	0.30	0.67 *	0.7 *	0.59 *		0.82 *	0.03	0.06	0.49 *	−0.21	0.02
Number of nursing homes/assisted living facilities	0.94 *	0.18	0.69 *	0.88 *	0.77 *	0.82 *		0.12	0.06	0.53 *	−0.24	0.07
Socioeconomic status SVI	0.20	0.48 *	0.12	0.23	0.27	0.03	0.12		0.52 *	0.42 *	0.5 *	0.93 *
Household characteristics SVI	0.09	0.13	−0.03	0.13	0.13	0.06	0.06	0.52 *		0.16	0.35 *	0.72 *
Minority and ethnicity status SVI	0.6 *	0.49 *	0.67 *	0.67 *	0.66 *	0.49 *	0.53 *	0.42 *	0.16		−0.13	0.37 *
Housing type and transportation SVI	−0.18	0.11	−0.34	−0.19	−0.14	−0.21	−0.24	0.5 *	0.35 *	−0.13		0.69 *
Overall SVI	0.15	0.4 *	0.03	0.20	0.24	0.02	0.07	0.93 *	0.72 *	0.37 *	0.69 *	

Pearson correlations with *p* < 0.05 were considered significant and are denoted by an *.

## Data Availability

The original data presented in the study are openly available on the Ohio Wastewater Monitoring Program website at https://odh.ohio.gov/know-our-programs/ohio-wastewater-monitoring-network/monitoring-data.

## Abbreviations

The following abbreviations are used in this manuscript:

InfA	Influenza A
InfB	Influenza B
SVI	Social Vulnerability Index
WWTP	Wastewater treatment plant
OWMN	Ohio Wastewater Monitoring Network
LOQ	Limit of quantification
ODHL	Ohio Department of Health Public Health Laboratory
BCoV	Bovine coronavirus
MGCPD	Million gene copies per person per day
IOP	Innovate Ohio Platform
ODRS	Ohio Disease Reporting System
NREVSS	National Respiratory and Enteric Virus Surveillance System
DHS	Department of Homeland Security
HIFLD	Homeland Infrastructure Foundation-Level Data
ATSDR	Agency for Toxic Substances and Disease Registry
CDC	Centers for Disease Control and Prevention
ODOT	Ohio Department of Transportation
SAS	Statistical Analysis Software
